# Supplementary figures and images for: Characterizing the distributions of IDO-1 expressing macrophages/microglia in human and murine brains and evaluating the immunological and physiological roles of IDO-1 in RAW264.7/BV-2 cells
Source: PLoS One. 2021 Nov 4;16(11):e0258204. doi: 10.1371/journal.pone.0258204 (PMC8568167; doi:10.1371/journal.pone.0258204)

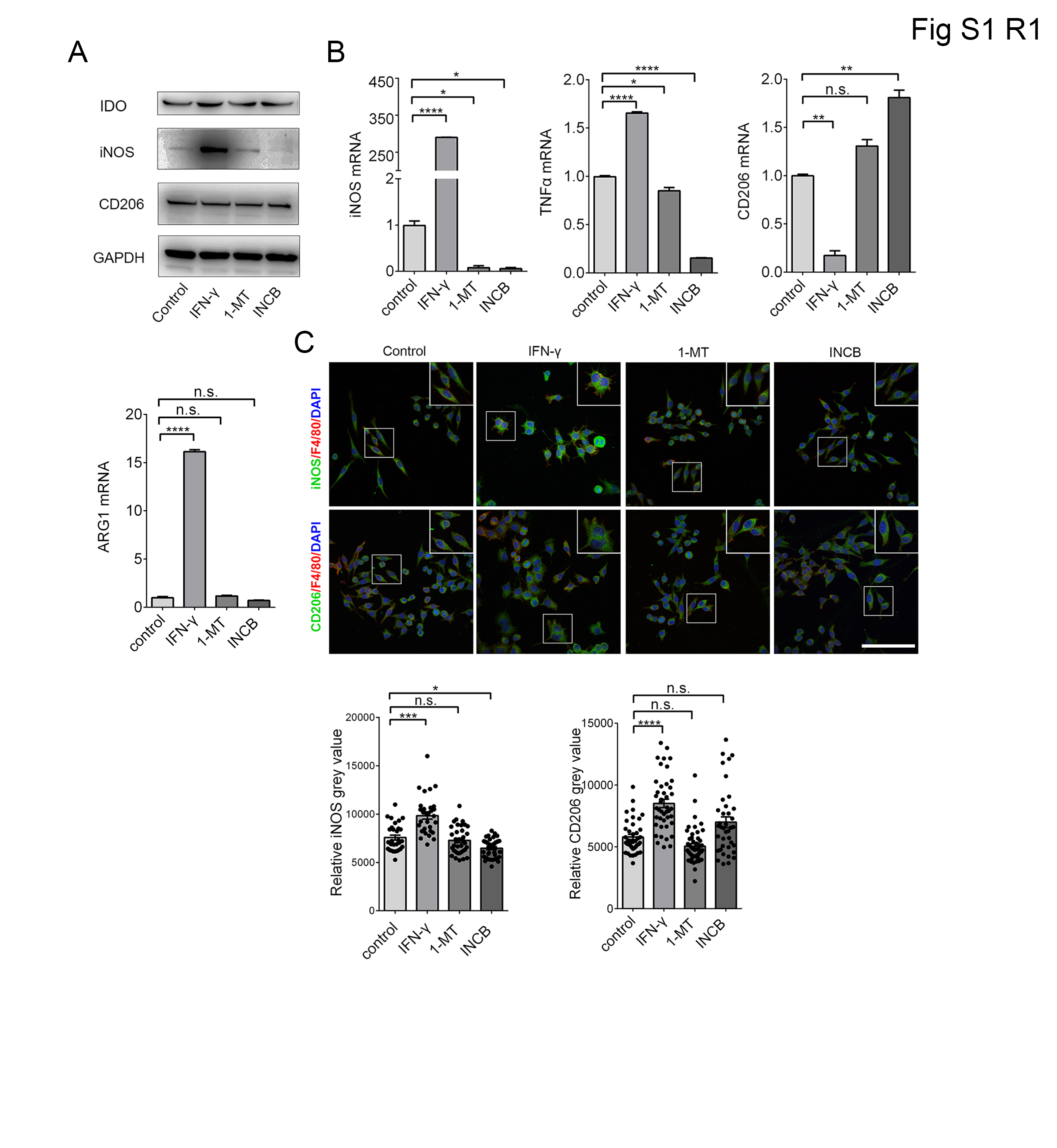

Supplement: S1 Fig — (A) The iNOS and CD206 expression in BV-2 cells treated with IFN-γ, 1-MT or INCB24360 for 24 h. (B) The transcription levels of iNOS, TNFα, CD206 and Arg1 in BV-2 cells treated with IFN-γ, 1-MT or INCB24360 for 24 h. (C) The immunostaining images of iNOS and CD206 in BV-2 cells treated with IFN-γ, 1-MT or INCB24360 for 24 h. The relative intensity of iNOS or CD206 in BV-2 cells after treatment with IFN-γ, 1-MT or INCB24360, which was measured by ImageJ software. n≥20. Scale bars, 100μm. One-way ANOVA; all data are expressed as the mean ± SEM. *, P<0.05, **, P<0.01; ns, no statistical difference. (TIF) [file pone.0258204.s001.tif]

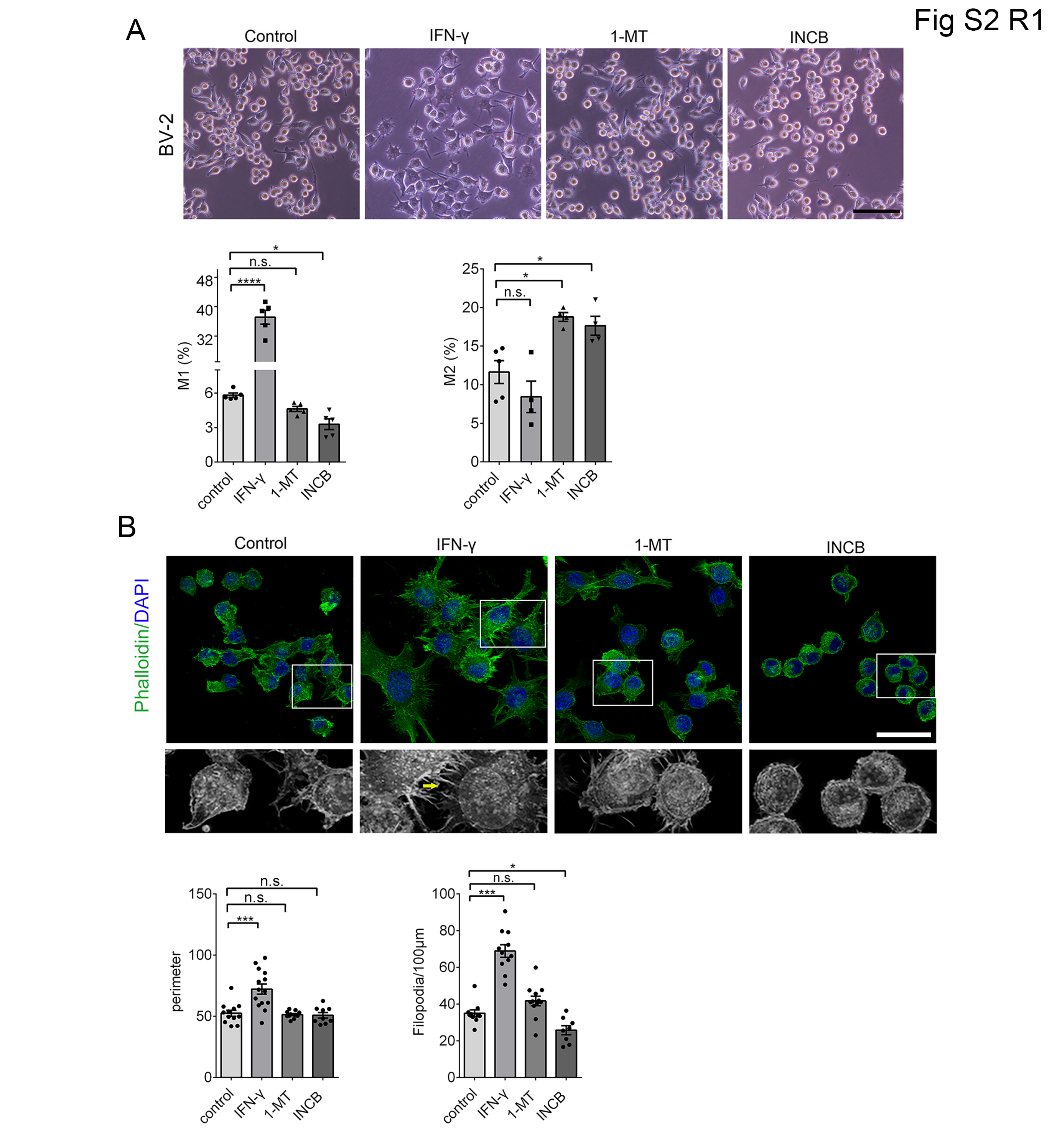

Supplement: S2 Fig — (A) The typical morphology of BV-2 cells treated with IFN-γ, 1-MT and INCB24360 for 24 h. The percentage of M1-like macrophage (ramified); M2-like macrophage (slender) in the control, IFN-γ, 1-MT and INCB24360 groups. N ≥ 5. Scale bars, 80μm. (B) The phalloidin Alexa-488 staining of BV-2 cells treated with IFN-γ, 1-MT or INCB24360 for 24 h. The cellular perimeters in the control, IFN-γ, 1-MT and INCB24360 groups. The density of the filopodia on the membrane of BV-2 cells in the control, IFN-γ, 1-MT and INCB24360 groups. n ≥10. Scale bars, 40μm. One-way ANOVA; all data are expressed as the mean ± SEM. *, P<0.05, **, P<0.01; ns, no statistical difference. (TIF) [file pone.0258204.s002.tif]

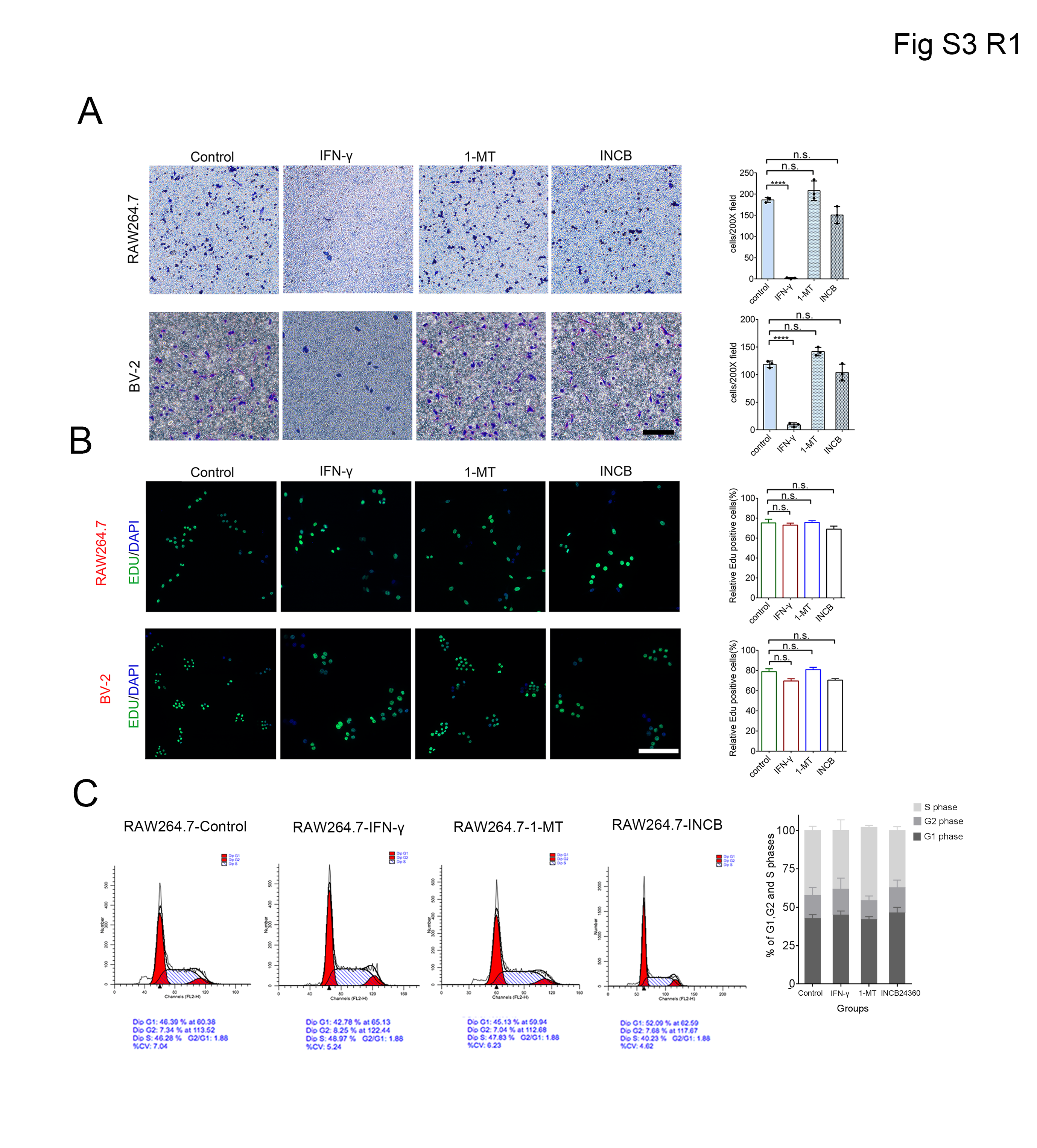

Supplement: S3 Fig — (A) The representative images of RAW264.7 or BV-2 cells treated with IFN-γ, 1-MT or INCB24360 in Transwell assay. Scale bars, 150μm. Quantifying migrating RAW264.7 or BV2 cells in Transwell assay. N = 3. Counts were done in ImageJ software. (B) The representative images of EDU assays in RAW264.7 or BV-2 cells treated with IFN-γ, 1-MT or INCB24360 for 24 h. Scale bars, 150μm. The percentage of the Edu- positive RAW264.7 or BV-2 cells treated with IFN-γ, 1-MT or INCB24360 for 24 h. N = 3, repeats. Count were done by ImageJ software. Scale bars, 150μm. One-way ANOVA; all data are expressed as the mean ± SEM. *, P<0.05, **, P<0.01; ns, no statistical difference. (C) The cell cycles of RAW264.7 cells treated with IFN-γ, 1-MT or INCB24360 for 24 h by Flow cytometry after PI staining. N = 4, repeats. (TIF) [file pone.0258204.s003.tif]

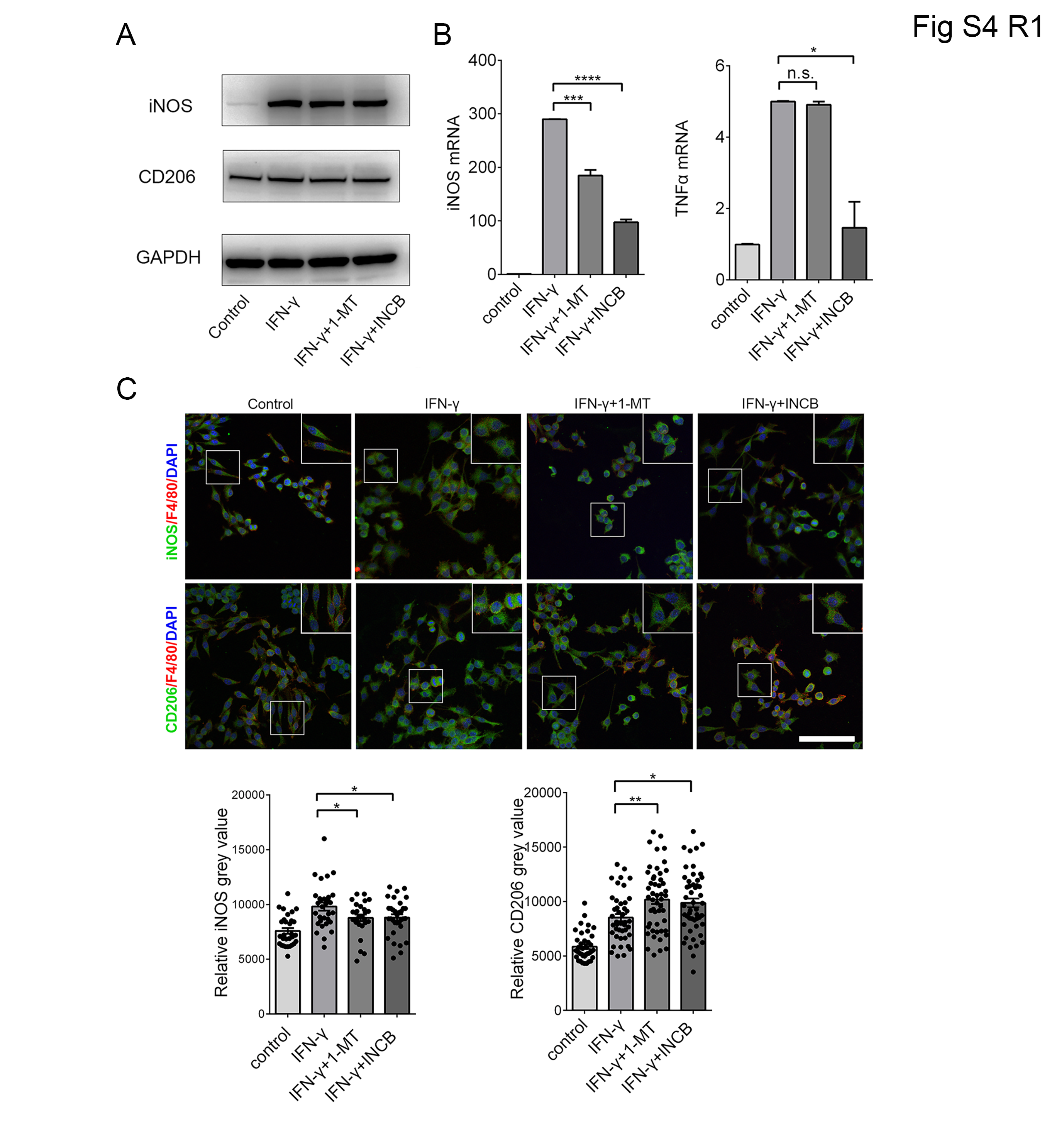

Supplement: S4 Fig — (A) iNOS and CD206 expression in BV-2 cells treated with IFN-γ, IFN-γ +1-MT or IFN-γ +INCB24360 for 24 h. (B) The transcription levels of iNOS and TNFα in BV-2 cells after treating with IFN-γ, IFN-γ +1-MT or IFN-γ +INCB24360 for 24 h. (C) The immunostaining images of iNOS and CD206 in BV-2 cells treated with IFN-γ, IFN-γ +1-MT or IFN-γ +INCB24360 for 24 h. iNOS or CD206 intensity measured by ImageJ software. Scale bars, 100μm. One-way ANOVA; all data are expressed as the mean ± SEM. *, P<0.05, **, P<0.01; ns, no statistical difference. (TIF) [file pone.0258204.s004.tif]

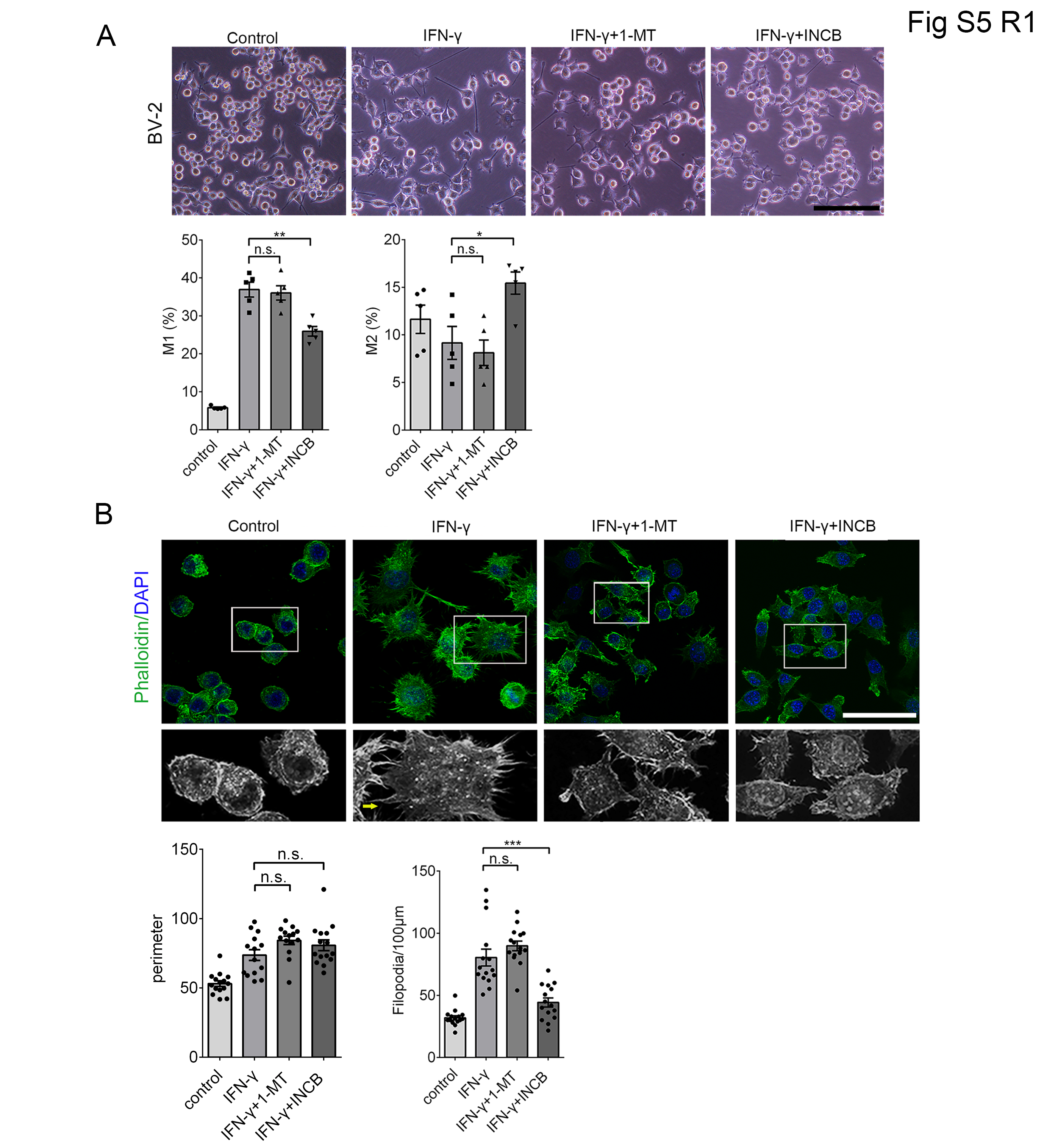

Supplement: S5 Fig — (A) The typical morphology of BV-2 cells after treatment with IFN-γ, IFN-γ+1-MT and IFN-γ + INCB24360; the percentage M1-like macrophage (ramified), and M2- like macrophage (slender) in the IFN-γ, IFN-γ +1-MT and IFN-γ +INCB24360 groups. N ≥ 5. Scale bars, 100μm. (B) The phalloidin Alexa-488 staining of BV2 cells treated with IFN-γ, IFN-γ+1-MT and IFN-γ + INCB24360 for 24 h. The cellular perimeters in the control, IFN-γ, IFN-γ+1-MT and IFN-γ +INCB24360 groups. The density of filopodia of BV2 cells in the control, IFN-γ, IFN-γ +1-MT and IFN-γ + INCB24360 groups. n≥10. Scale bars, 50μm. One-way ANOVA; all data are expressed as the mean ± SEM. *, P<0.05, **, P<0.01; ns, no statistical difference. (TIF) [file pone.0258204.s005.tif]

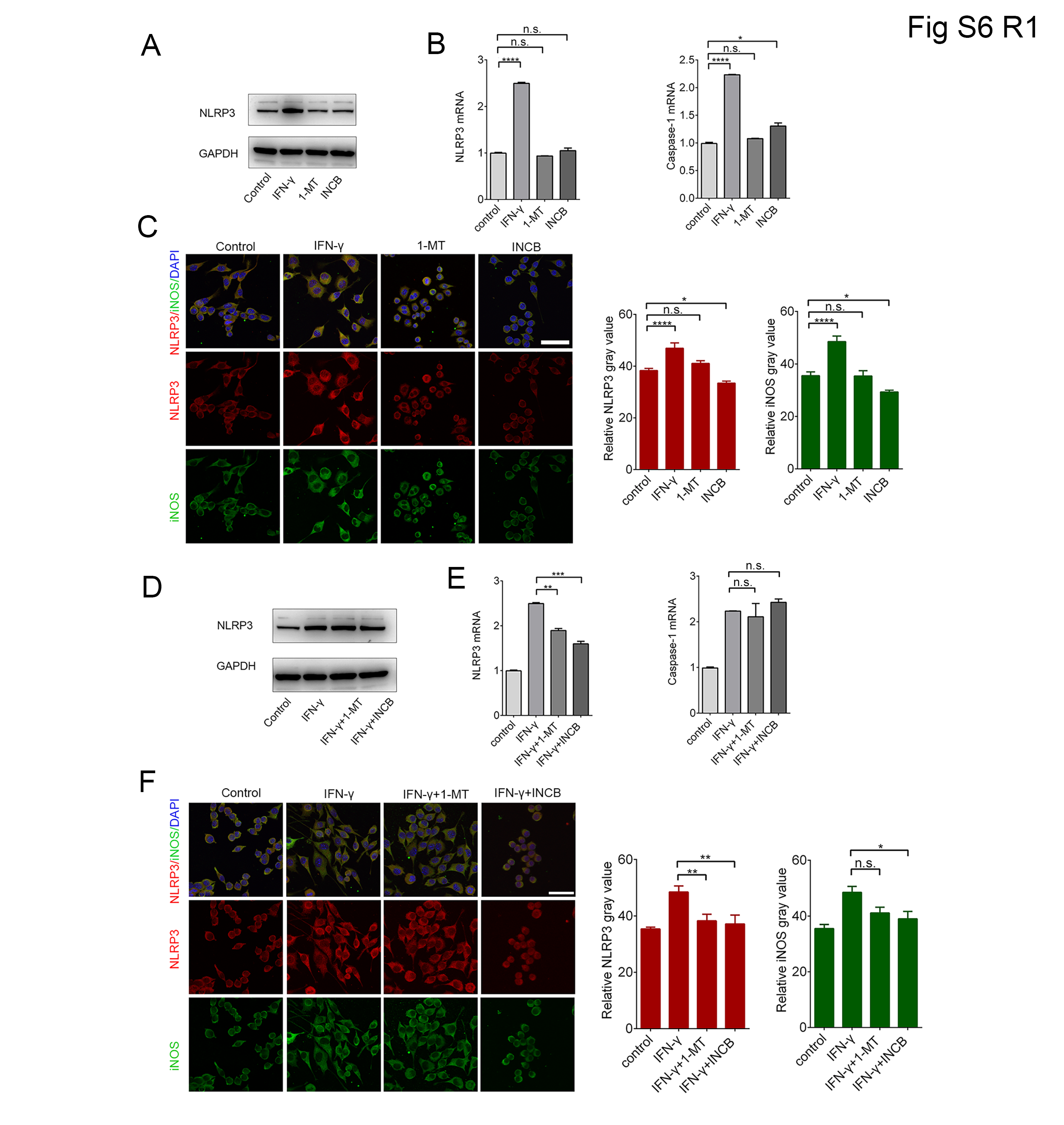

Supplement: S6 Fig — (A) NLRP3 and caspase-1 expression in BV-2 cells after treated with IFN-γ, 1-MT and INCB for 24 h. (B) The transcription levels of NLRP3 and caspase-1 in BV-2 cells treated with IFN-γ, 1-MT and INCB for 24 h. (C) The immunostaining images of NLRP3 and iNOS in BV-2 cells treated with IFN-γ, 1-MT and INCB24360 for 24 h. NLRP3 or iNOS intensity measured by ImageJ. n≥20. Scale bars, 50μm. (D) NLRP3 and caspase-1 expression in BV-2 cells treated with IFN-γ, IFN-γ +1-MT or IFN-γ +INCB24360 for 24 h. (E) The transcription levels of NLRP3 and caspase-1 in BV-2 cells treated with IFN-γ, IFN-γ +1-MT or IFN-γ +INCB24360 for 24 h. (F) The immunostaining images of NLRP3 and iNOS in BV-2 cells treated with IFN-γ, IFN-γ +1-MT or IFN-γ +INCB for 24 h. NLRP3 or iNOS intensity measured by ImageJ. Scale bars, 50μm. One-way ANOVA; all data are expressed as the mean ± SEM. *, P<0.05, **, P<0.01; ns, no statistical difference. (TIF) [file pone.0258204.s006.tif]

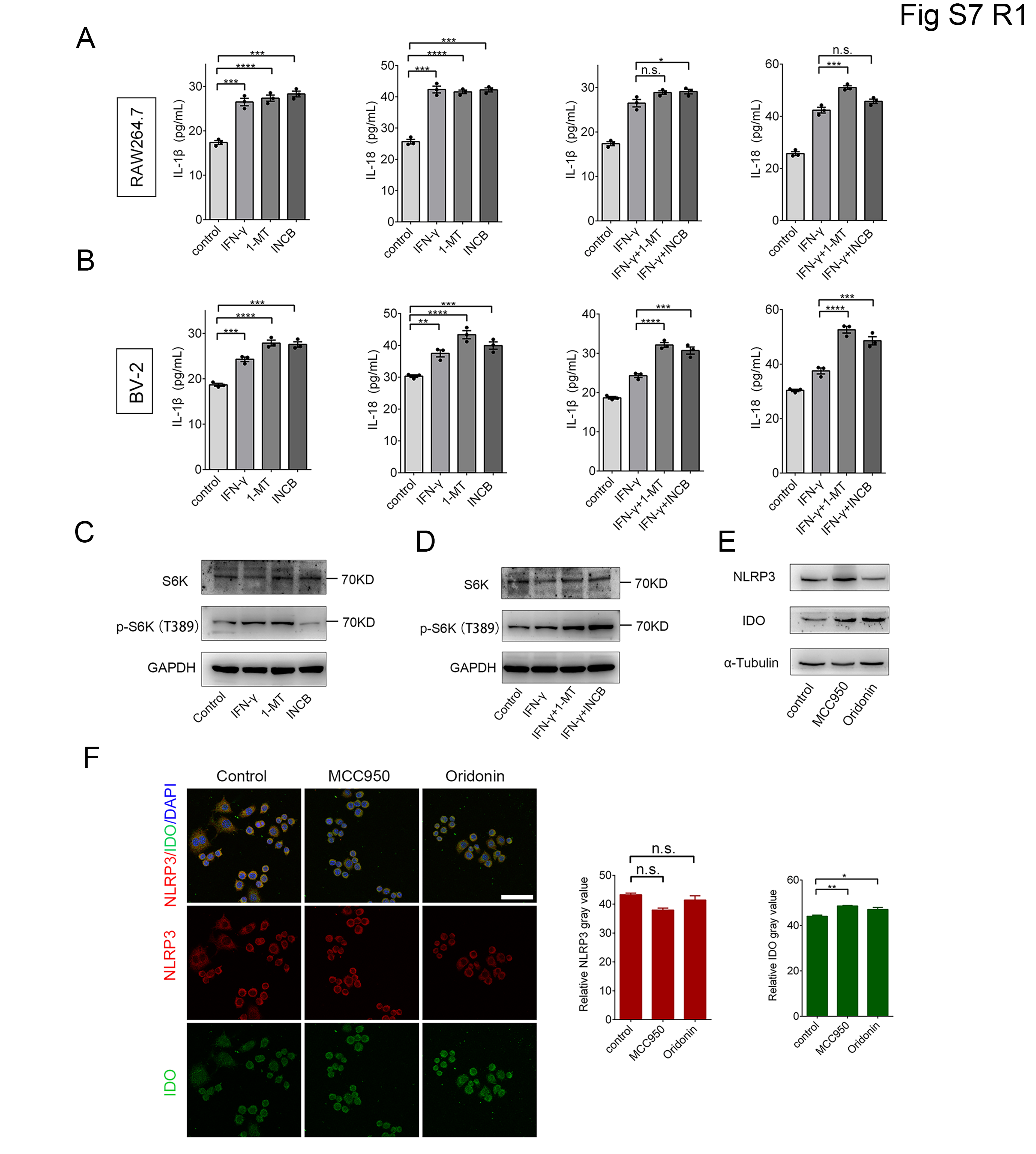

Supplement: S7 Fig — (A) IL-1β and IL18 secretion Levels in RAW264.7 cells (with ELISA) treated with IFN-γ, 1-MT or INCB24360 for 24 h. IL-1β and IL18 secretion Levels in RAW264.7 cells (with ELISA) treated with IFN-γ, IFN-γ+1-MT or IFN-γ +INCB24360 for 24 h. (B) IL-1β and IL18 secretion Levels in BV2 cells (with ELISA) treated with IFN-γ, 1- MT or INCB24360 for 24 h. IL-1β and IL18 secretion Levels in RAW264.7 cells (with ELISA) treated with IFN-γ, IFN-γ+1-MT or IFN-γ +INCB24360 for 24 h. (C, D) S6K and p-S6K protein levels in RAW264.7 treated with IFN-γ, 1-MT or INCB24360 for 24 h. S6K and p-S6K protein levels in RAW264.7 treated with IFN-γ, IFN-γ+1-MT or IFN-γ +INCB24360 for 24 h. (E) The changes of NLRP3 and IDO expression in RAW264.7 treated by MCC950 and Oridonin for 24 h. (F) The representative immunostaining results of NLRP3 and IDO RAW264.7 cells treated with MCC950 and IDO for 24 h. The relative levels of NLRP3 or IDO intensity in RAW264.7 cells after drug treatment, measured by image J. Scale bars, 50μm. One-way ANOVA; all data are expressed as the mean ± SEM. *, P<0.05, **, P<0.01; ns, no statistical difference. (TIF) [file pone.0258204.s007.tif]
