# Supplementary material for: Characterizing the distributions of IDO-1 expressing macrophages/microglia in human and murine brains and evaluating the immunological and physiological roles of IDO-1 in RAW264.7/BV-2 cells
Source: PLoS One. 2021 Nov 4;16(11):e0258204. doi: 10.1371/journal.pone.0258204 (PMC8568167; doi:10.1371/journal.pone.0258204)

Figure2

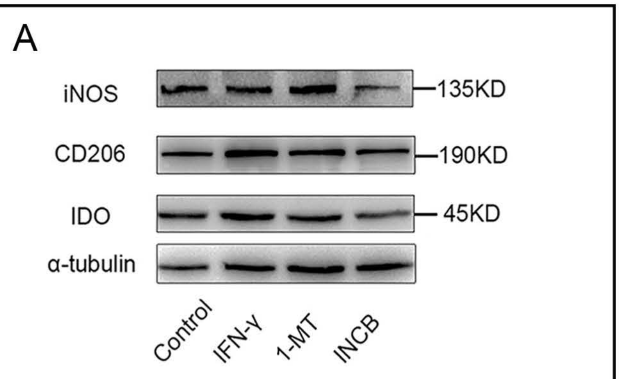


original image

iNOS

(1)

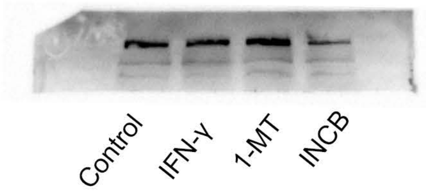


(2)

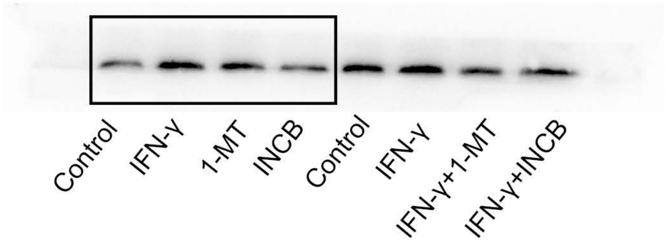


(3)

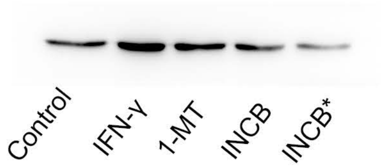


* means repeats

CD206

(1)

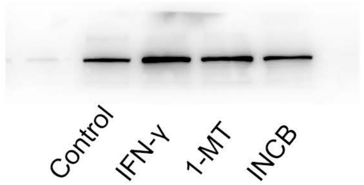


(2)

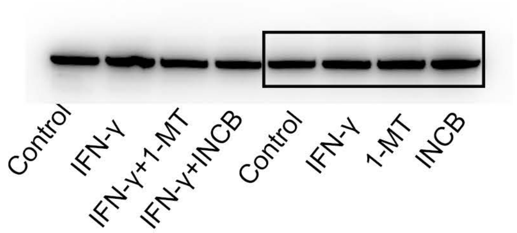


(3)

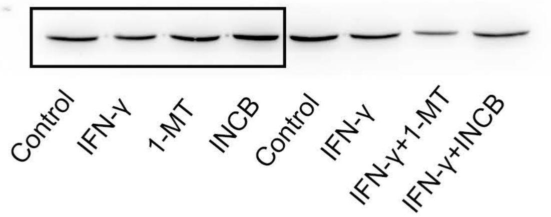

IDO

(1)

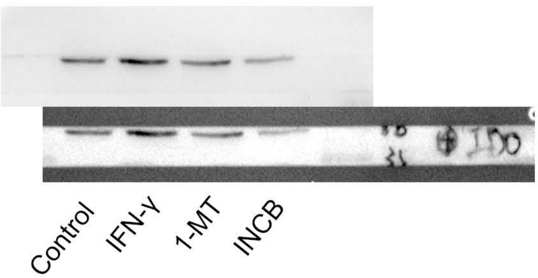
Control
IFN-γ
1-MT
INCB

(2)

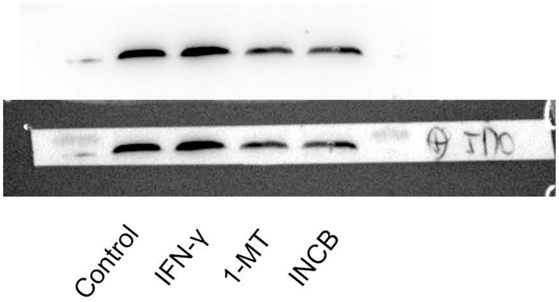
Control
IFN-γ
1-MT
INCB

(3)

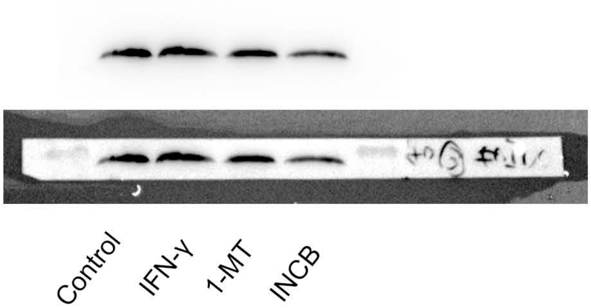
Control
IFN-γ
1-MT
INCB

Figure5

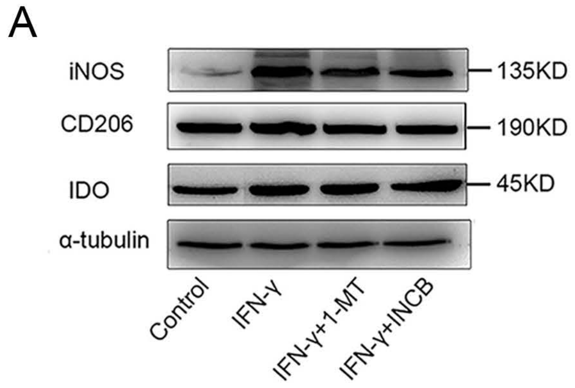


original image

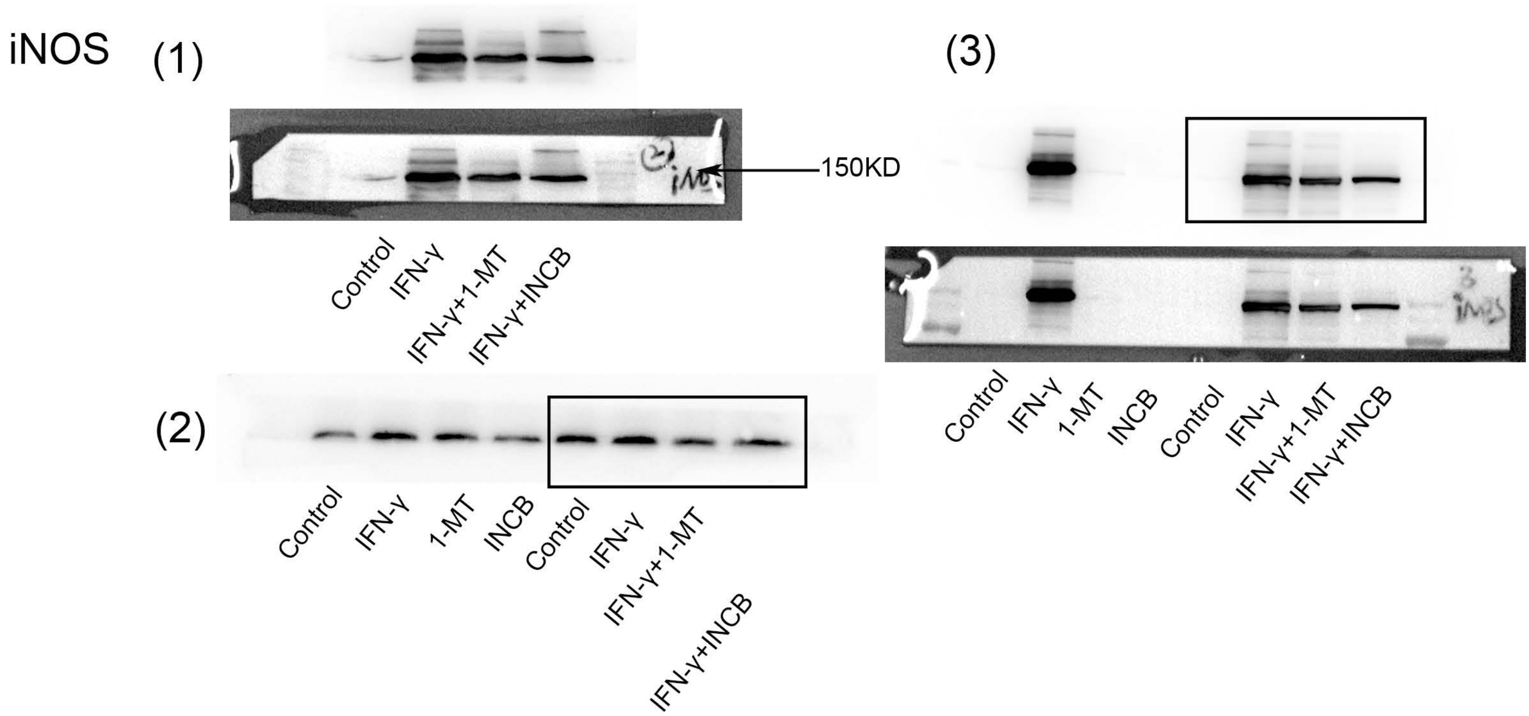


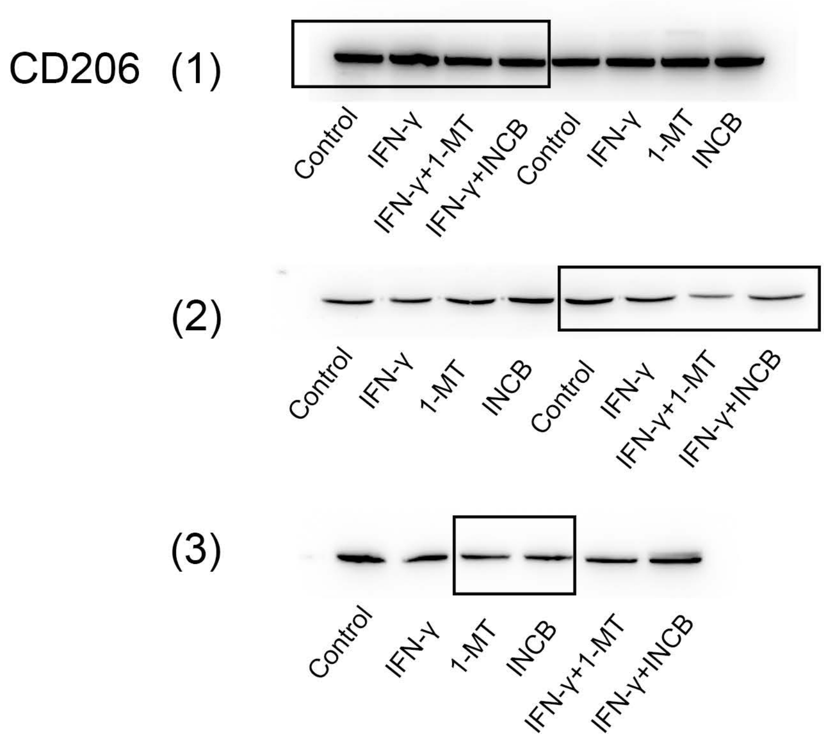

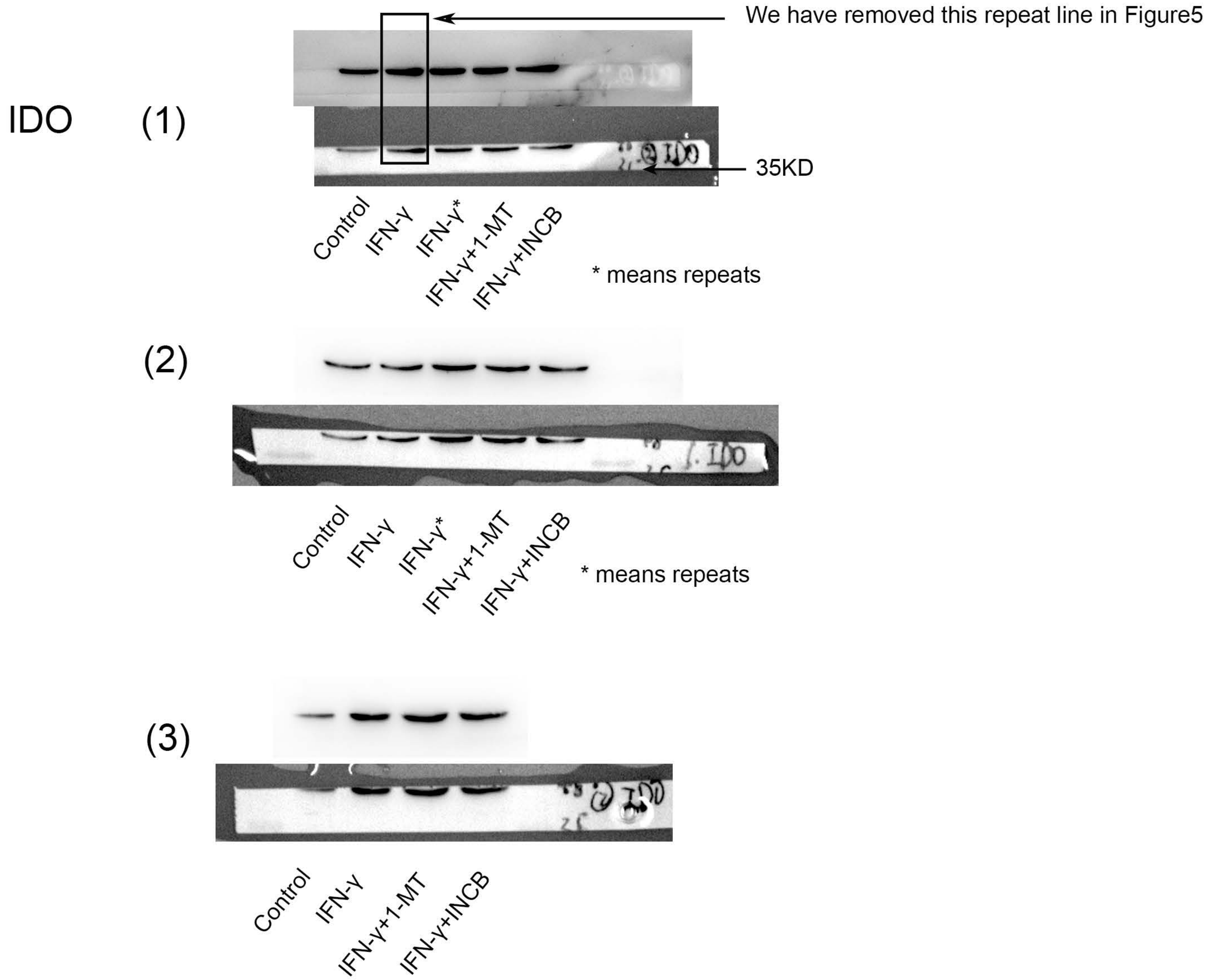
IDO
(1)
We have removed this repeat line in Figure5
35KD
Control
IFN-γ
IFN-γ*
IFN-γ+1-MT
IFN-γ+INCB
* means repeats
(2)
Control
IFN-γ
IFN-γ*
IFN-γ+1-MT
IFN-γ+INCB
* means repeats
(3)
Control
IFN-γ
IFN-γ+1-MT
IFN-γ+INCB

Figure8

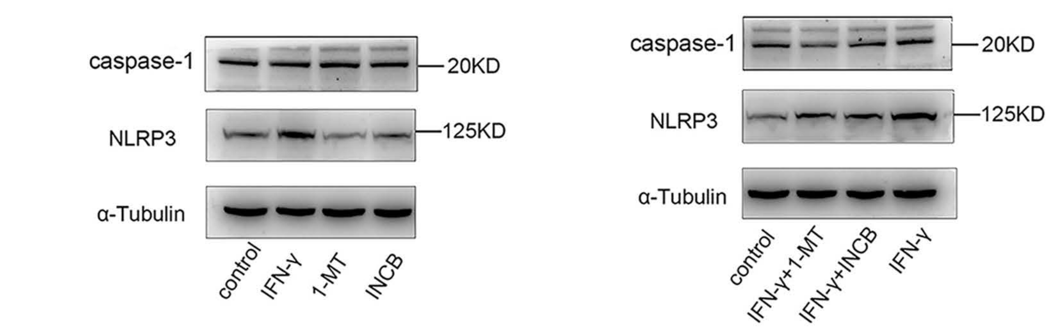


original image

NLRP3

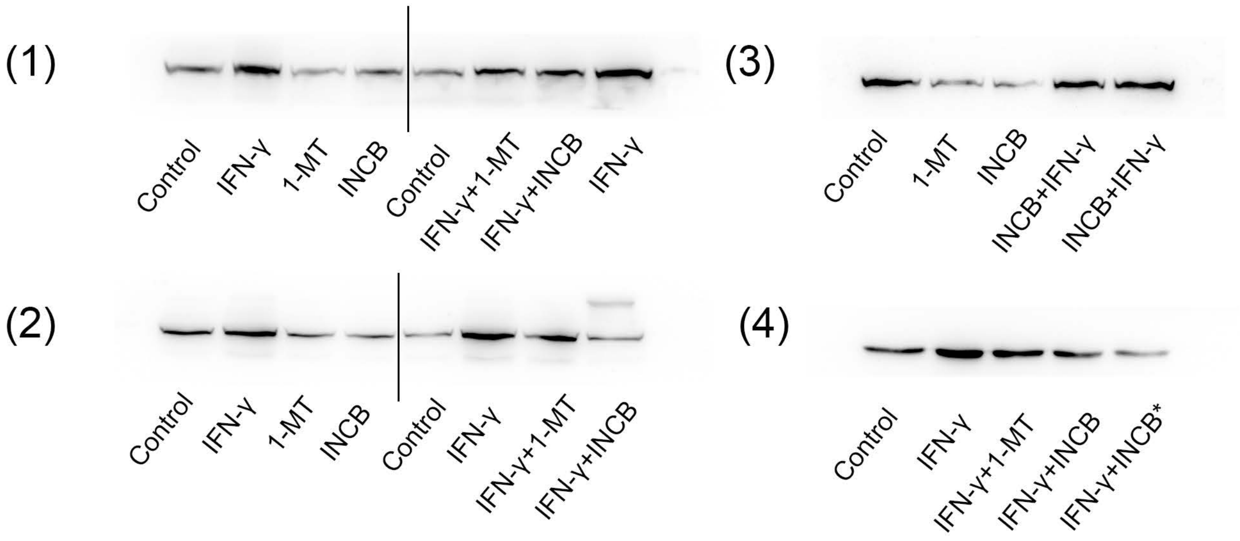


Caspase-1

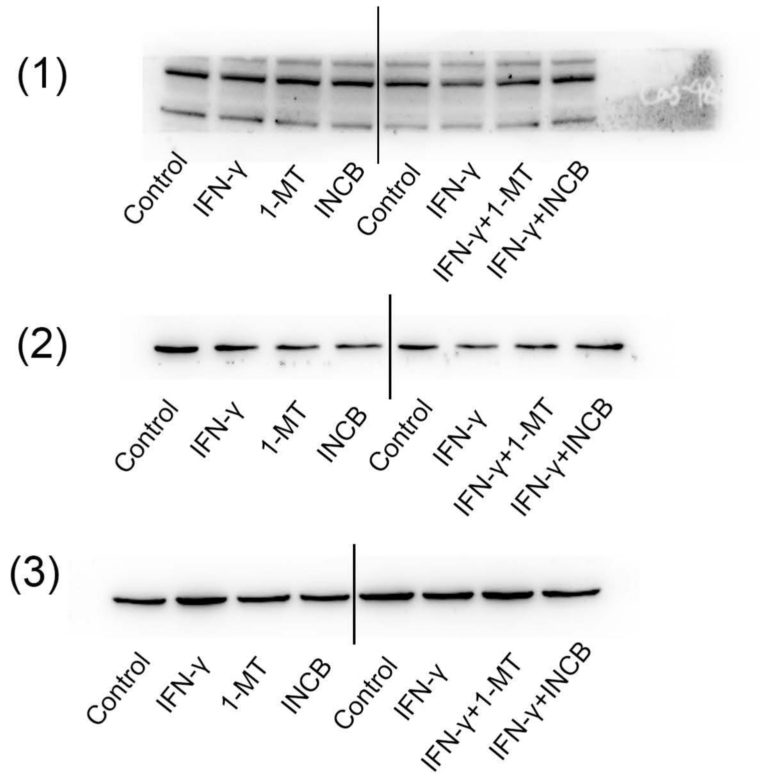

Supplement: S1 Raw images — (PDF) [file pone.0258204.s008.pdf]
