# Supplementary material for: Characterizing the distributions of IDO-1 expressing macrophages/microglia in human and murine brains and evaluating the immunological and physiological roles of IDO-1 in RAW264.7/BV-2 cells
Source: PLoS One. 2021 Nov 4;16(11):e0258204. doi: 10.1371/journal.pone.0258204 (PMC8568167; doi:10.1371/journal.pone.0258204)
Supplement: Attachment — Submitted filename: Response to Reviewers.docx [file pone.0258204.s010.docx]

Reviewers' comments:

Reviewer's Responses to Questions

Comments to the Author

1. If the authors have adequately addressed your comments raised in a previous round of review and you feel that this manuscript is now acceptable for publication, you may indicate that here to bypass the “Comments to the Author” section, enter your conflict of interest statement in the “Confidential to Editor” section, and submit your "Accept" recommendation.

Reviewer #1: All comments have been addressed

Thanks again.

Reviewer #2: All comments have been addressed

Thanks again.

Reviewer #3: All comments have been addressed

Thanks again.

2. Is the manuscript technically sound, and do the data support the conclusions?

The manuscript must describe a technically sound piece of scientific research with data that supports the conclusions. Experiments must have been conducted rigorously, with appropriate controls, replication, and sample sizes. The conclusions must be drawn appropriately based on the data presented.

Reviewer #1: Yes

Reviewer #2: Yes

Reviewer #3: Yes

3. Has the statistical analysis been performed appropriately and rigorously?

Reviewer #1: Yes

Reviewer #2: Yes

Reviewer #3: Yes

4. Have the authors made all data underlying the findings in their manuscript fully available?

The PLOS Data policy requires authors to make all data underlying the findings described in their manuscript fully available without restriction, with rare exception (please refer to the Data Availability Statement in the manuscript PDF file). The data should be provided as part of the manuscript or its supporting information, or deposited to a public repository. For example, in addition to summary statistics, the data points behind means, medians and variance measures should be available. If there are restrictions on publicly sharing data—e.g. participant privacy or use of data from a third party—those must be specified.

Reviewer #1: Yes

Reviewer #2: Yes

Reviewer #3: Yes

5. Is the manuscript presented in an intelligible fashion and written in standard English?

PLOS ONE does not copyedit accepted manuscripts, so the language in submitted articles must be clear, correct, and unambiguous. Any typographical or grammatical errors should be corrected at revision, so please note any specific errors here.

Reviewer #1: Yes

Reviewer #2: Yes

Reviewer #3: Yes

6. Review Comments to the Author

Please use the space provided to explain your answers to the questions above. You may also include additional comments for the author, including concerns about dual publication, research ethics, or publication ethics. (Please upload your review as an attachment if it exceeds 20,000 characters)

Reviewer #1: The authors have made all changes necessitated by the first round of review process. All additional data presented by the authors fully support the authors conclusion. I have no further comments or concerns.

Dear reviewer #1: Thanks again. Your comments are helpful to better our manuscript.

Reviewer #2: The authors addressed the main concerns. Although this paper could be supplemented with stronger evidences, the theory presented opens new scopes in this field for further explorations.

Dear reviewer #2: Thanks for the insightful comments about our manuscript. Your concerns about this paper are also our primary concern in the future. After this pandemic is over, we will address the immunological role of IDO+ macrophage/microglia in meninges and perivascular space, even the immune homeostasis in the parenchyma in our subsequent work. We are preparing more in vivo experiments to address your and our concerns about IDO-1+ macrophages/microglia in the brain.

Reviewer #3: The authors have done a good job of addressing the questions raised by the reviewers. However, I felt the authors were adamant and stubborn in their responses. I wish they could use their wisdom to respond the questions raised by the reviewers.

Dear Dr. Mallikarjun Patil:

Thanks for taking the time to review our manuscript. In this specific season, we try our best to revise our manuscript. The availability of core facilities, especially FACS, is limited because of some restrictions and regulations. As non-native speakers of English, it is a little difficult for us to fluently and smartly communicate with reviewers or editors as a native speakers in the scientific community. In the future, we will try our best to improve our communication skills in the scientific literature, and you will have a chance to a better work about meningeal immunity soon. If some responses are inappropriate, it might be caused by our communication skills but not our true intention. Thanks for raising these questions. We will try our best to respond to the comments of reviewer and editor with wisdom in the future.

7. PLOS authors have the option to publish the peer review history of their article (what does this mean?). If published, this will include your full peer review and any attached files.

If you choose “no”, your identity will remain anonymous but your review may still be made public.

Do you want your identity to be public for this peer review? For information about this choice, including consent withdrawal, please see our Privacy Policy.

Reviewer #1: No

Reviewer #2: No

Reviewer #3: Yes: Mallikarjun Patil

Thank again for reviewers and editor taking the time to review our manuscript. If you have any further questions or concerns about this manuscript, we are willing to respond.

We are looking forward to hear from you soon.

On the behalf of all authors

Dr. Hexige Saiyin

The School of Life Science

Fudan University

Email: saiyin@fudan.eud.cn
